# Urban Air Pollution and Plant Tolerance: Omics Responses to Ozone, Nitrogen Oxides, and Particulate Matter

**DOI:** 10.3390/plants13152027

**Published:** 2024-07-24

**Authors:** Maria Luisa Antenozio, Cristina Caissutti, Francesca Maria Caporusso, Davide Marzi, Patrizia Brunetti

**Affiliations:** 1 Research Institute on Terrestrial Ecosystems (IRET), National Research Council of Italy (CNR), 00015 Monterotondo, Italy; marialuisa.antenozio@cnr.it (M.L.A.); cristina.caissutti@iret.cnr.it (C.C.); caporusso.1904050@studenti.uniroma1.it (F.M.C.); 2 Department of Biology and Biotechnologies ‘Charles Darwin’ (BBCD), Sapienza University of Roma, 00185 Roma, Italy; 3 National Biodiversity Future Center (NBFC), 90133 Palermo, Italy

**Keywords:** plant species, ozone, nitrogen dioxide, particulate matter, photosynthesis, reactive oxygen species, phenylpropanoids, transcription factors

## Abstract

Urban air pollution is a crucial global challenge, mainly originating from urbanization and industrial activities, which are continuously increasing. Vegetation serves as a natural air filter for air pollution, but adverse effects on plant health, photosynthesis, and metabolism can occur. Recent omics technologies have revolutionized the study of molecular plant responses to air pollution, overcoming previous limitations. This review synthesizes the latest advancements in molecular plant responses to major air pollutants, emphasizing ozone (O_3_), nitrogen oxides (NO_X_), and particulate matter (PM) research. These pollutants induce stress responses common to other abiotic and biotic stresses, including the activation of reactive oxygen species (ROSs)-scavenging enzymes and hormone signaling pathways. New evidence has shown the central role of antioxidant phenolic compound biosynthesis, via the phenylpropanoid pathway, in air pollution stress responses. Transcription factors like WRKY, AP2/ERF, and MYB, which connect hormone signaling to antioxidant biosynthesis, were also affected. To date, research has predominantly focused on laboratory studies analyzing individual pollutants. This review highlights the need for comprehensive field studies and the identification of molecular tolerance traits, which are crucial for the identification of tolerant plant species, aimed at the development of sustainable nature-based solutions (NBSs) to mitigate urban air pollution.

## 1. Introduction

Air pollution is defined as the pollution of indoor or outdoor environments caused by chemical, physical, or biological factors that alter the natural characteristics of the atmosphere [1]. Since the 19th century, the global trend toward urbanization and industrialization has been accompanied by a dramatic detrimental increase in air pollution [2]. The latest release from the World Health Organization’s (WHO) Ambient Air Quality Database indicates that almost the entire global population (99%) breathes air that exceeds the quality limits [1,3], which leads to augmented health risks. The adverse effects of air pollution on human health are associated with increased mortality linked to cardiovascular and respiratory diseases, including infections related to the respiratory tract [1]. 

Air pollution is a mixture of hazardous substances from both anthropic activity and natural sources. Traffic and mobility, industrial production, and heating systems are the main sources of urban air pollution [1]. 

**Figure 1:**
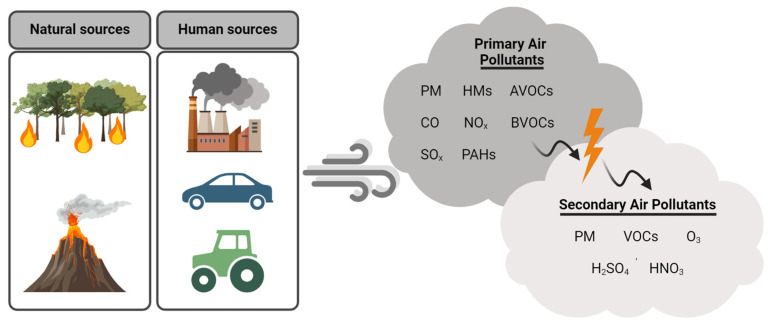
Representative image of the main natural and human sources of primary and secondary air pollutants. Arrows in the clouds indicate the photochemically-driven generation of secondary air pollutants from the primary ones. In detail symbols indicate: PM, particulate matter; HMs, heavy metals; VOCs, volatile organic compounds; CO, carbon monoxide; NOx, nitrogen oxides; SOx, sulfur oxides; PAHs, polycyclic aromatic hydrocarbons; O_3_, ozone; H_2_SO_4_, sulfuric acid; HNO_3_, nitric acid.

A general classification divides air pollutants into primary and secondary pollutants, depending on whether they are directly discharged into the atmosphere or whether they derive from the interaction between primary pollutants and other molecules present in the air. Primary pollutants include gaseous molecules, such as sulfur oxides (SO_X_) and nitrogen oxides (NO_X_), which are mainly generated from the combustion of different substrates, for instance fossil fuels. Further primary pollutants include particulate matter (PM), a heterogeneous solid–liquid mixture composed of various particles of different size and origins, which has been shown to be closely related to health issues in cities [4]. Sulphur dioxide (SO_2_), NO_X_, and PM are among the main pollutants found in urban areas. Volatile organic compounds (VOCs) are primary pollutants deriving from both human activities (anthropogenic VOCs-AVOCs) and natural sources (biogenic VOCs-BVOCs). The former originate from the transport industry, the use of solvent chemicals, production activities, storage, and combustion processes, while the latter include molecules emitted by plants for long-range communication, such as isoprene, monoterpenes, and sesquiterpenes, which significantly impact air quality [5,6,7] (Figure 1).

Sunlight triggers photochemical reactions between primary pollutants in the atmosphere, such as carbon monoxide (CO), volatile organic compounds (VOCs), and NOx, resulting in the production of secondary pollutants, such as ground-level ozone (O_3_), sulfuric acid (H_2_SO_4_), nitric acid (HNO_3_), secondary VOCs, and PM, increasing the complexity of air pollution and resulting in acid rain formation, further affecting air quality and human health [1,3] (Figure 1). In addition, BVOCs participate in the production of secondary organic aerosols (SOAs) upon oxidation in the atmosphere, releasing into the air organic products containing single or multiple oxygenated functional groups like, –CHO, –OH, >CO, –NO_2_, –COOONO_2_, –COOH, –OOH, and –COOOH, which may give rise to other reactions, producing further pollution [8]. 

According to the aerodynamic diameter, PM is divided into coarse particles (≤10 μm, PM_10_), fine particles (≤2.5 μm, PM_2.5_), and ultrafine particles (≤0.1 μm, PM_0.1_) [9]. Among the different air pollutants, PM is the most widespread, due to its longer atmospheric duration and its presence in both urban and rural areas [1,3].

Particulate matter can contain different chemical elements, including heavy metals (HMs), such as lead (Pb), copper (Cu), cadmium (Cd), chromium (Cr), mercury (Hg), arsenic (As), and antimony (Sb), mainly derived from agriculture, industrial activities, and traffic emissions [10,11]. For instance, the analysis of road dust collected in urban parks in Beijing (China) revealed the presence of nickel (Ni), zinc (Zn), Cr, Cu, Cd, and Pb at a concentration of 25.97, 219.20, 69.33, 72.13, 0.64, and 201.82 mg kg^−1^, respectively [12]. Furthermore, an extensive study conducted in 210 locations in 16 countries showed that the main components of PM_2.5_ were sulphate, nitrate, ammonium, black carbon, organic carbon, mineral dust, and sea salt, which are associated with increased health risks [13]. 

Diesel exhaust (DE) emissions are a complex mixture of gases and fine particles emitted from diesel engines in vehicles, as well as off-road diesel engines used in agricultural, maintenance, and construction equipment [14]. Diesel exhaust emissions significantly contribute to urban pollution and health-related diseases, since it contains various toxic chemicals, including CO, CO_2_, SO_2_, NO_X_, aldehydes (formaldehyde, acrolein, acetaldehyde), benzene, polycyclic aromatic hydrocarbons (PAHs), and PM [a partial list of the chemicals associated with diesel exhaust emissions can be found at: https://www.osha.gov/diesel-exhaust/chemical (accessed on 23 July 2024)]. 

Urban and rural vegetation acts as a natural sponge, absorbing pollutants from the air. The morphological and physiological features of plants, like the extended leaf area and the microstructure on the leaf surface, promote PM deposition on leaves [15,16]. However, air pollution may affect plant health, inducing different phytotoxic responses depending on the physical and chemical features of the pollutant. Foliar injury, pigment loss, premature senescence, and decreased photosynthetic/growth rates, are the main symptoms induced by O_3_ and NO_2_ in plants. Particulate matter accumulation on leaves can alter their optical properties, affecting the absorption and reflection of photosynthetically active radiation (PAR), clogging stomata, reducing photosynthesis and respiration, and, ultimately, decreasing plant growth and yields due to disrupted stomatal movement [17,18]. Depending on the physical and chemical composition, PM may induce different phytotoxic responses in plants. Additionally, PM can increase the leaf’s surface temperature and indirectly impact plant health by disturbing beneficial microbial communities in the phyllosphere and leaf endosphere, thus negatively affecting plant growth and health [19,20].

Air pollution may also affect plant growth, inducing a plethora of stress responses, including oxidative stress, including ROS burst, which is among the most common effects caused by air pollution and triggers the deregulation of reactive oxygen species (ROSs)-scavenging enzymes, such as catalase (CAT), superoxide dismutase (SOD), ascorbate peroxidase (APX), and peroxidase (POD). At the same time, oxidative stress is generally counteracted by the enhanced biosynthesis of antioxidant molecules, namely flavonoids derived from the phenylpropanoid pathway [21]. 

So far, most of the research has been conducted in laboratory conditions, focusing on the effects of individual pollutants and rarely analyzing the impact of their combined presence. To unveil the role of the actors regulating the responses to air pollution at the molecular level, mutant lines in the model plant species *Arabidopsis thaliana* [22] have been analyzed after treatment with O_3_ and NO_2_. The null mutation in the genes encoding for enzymes involved in photorespiration, such as *glycolate oxidases GOX1* and *GOX2*, *glutmate:glyoxylate aminotransferase* (*GGAT*), and *NADH-dependent hydroxypyruvate reductase* (*HPR*), triggered enhanced sensitivity to O_3_ [23]. Conversely, the null mutation in the gene encoding for *ethylene insensitive 2* (*ein2-1*) showed resistance traits upon NO_2_ fumigation, such as enhanced activity of SOD, POD, and CAT of about 39%, 92%, and 11%, compared to wild-type plants [24]. Attempts to produce “air-pollutant-philic” plants through genetic engineering have mainly failed, but have led to the discovery of novel mechanisms regarding plant tolerance to NO_2_ [25]. On the other hand, the production of transgenic trees with reduced BVOC emissions, such as the gray poplar species *Populus* × *canescens* transformed for its RNA interference against the *isoprene synthase* gene, has led to the production of plants with reduced BVOC emissions, while maintaining their photosynthetic and growth performance in comparison to that of control plants [26]. In addition, transgenic poplar (*Populus tremula* × *Populus alba*) plants overexpressing *cytochrome P450 2E1*, which is involved in the metabolism of different halogenated compounds, demonstrated a superior level of removal of pollutants, such as trichloroethylene, vinyl chloride, carbon tetrachloride, benzene, and chloroform, from air [27]. However, so far, research on transgenic plants with increased tolerance or accumulation of air pollution is still limited, hindering the application of such plants to restore air quality.

A few recent studies have investigated the effect of air pollution on the molecular mechanisms of plants by performing the analysis directly on vegetation grown in urbanized and polluted areas. Thus, new data are expected, at both the laboratory and field scale, to unveil the effects induced by the combination of various air pollutants and the related plant molecular responses that could drive adaptation to air pollution. Several plant species have evolved tolerance mechanisms toward air pollution, paving the way for an assigned application of sustainable nature-based solutions (NBS) to mitigate the negative effects of air pollution in urban areas. Indeed, the implementation of vegetation in the form of green infrastructure (GI) is a promising strategy that has been recently implemented worldwide [28]. GI takes advantage of the ability of plants to absorb, accumulate, and degrade atmospheric pollutants [29]. Therefore, the study of the molecular mechanisms activated by plants in response to air pollution and the identification of the molecular tolerance traits exhibited by different plant species, become of utmost importance in order to provide refined tools to support the application of GI.

The adaptation of plants to air pollution involves a complex regulatory network of phytohormone signaling pathways, such as that of abscisic acid (ABA), jasmonic acid (JA), salicylic acid (SA), ethylene, cytokinin, brassinosteroids (BRs), and auxin, which are generally involved in plant responses to abiotic and biotic stress [30,31,32,33,34]. In addition, several transcription factors (TFs) involved in stress responses, such as WRKY (named after the WRKYGQK heptapeptide at the N-terminal end), MYB (first identified as an oncogene derived from the avian myeloblastosis virus), and APETALA2/ethylene responsive factor (APA2/ERF), are also involved in air pollution responses, activating gene expression and bridging the signaling pathway of different hormones, like ABA and JA, induced by stress [35]. Amino acid metabolism also has a primary role in stress responses, indeed phenylalanine is used as a substrate by enzymes involved in the production of phenylpropanoids, like phenylalanine ammonia-lyase (PAL) and cinnamate 4-hydroxylase (C4H). Downstream of PAL and C4H, chalcone synthase (CHS) catalyzes the first step in the flavonoid pathway. Interestingly, in-depth analysis of gene expression has uncovered recurring patterns, outlining the presence of conserved mechanisms activated in plants to cope with air pollution. 

This review focuses on the latest findings from studies on the effects of air pollution on plant growth and molecular responses induced in plant species. Compared to recent reviews that mainly deal with the physiological and biochemical effects triggered by air pollution in plants [36,37,38,39], this review focuses on recent omics data to describe new findings regarding the regulation of transcriptomes, proteomes, and metabolomes of plants in response to air pollution. The bibliographic research highlights that: (1) O_3_, NO_x_, PM_2.5_, PM_10_, and HMs are the most studied pollutants; (2) most of the studies are based on fumigation experiments; and (3) field analysis is limited so far. However, the small amount of evidence and the variety of experimental approaches result in a matrix of fragmented information that needs to be further investigated in order to fill the gaps. In addition to laboratory scale experiments, future studies should include open field trials, which could lead to the identification of deregulated pathways and tolerance traits in different plant species and within the same species. 

## 2. Air Pollution Induces Similar but Not Overlapping Molecular Responses in Plants

In the past decades, several studies have attempted to investigate the molecular responses induced in plants by air pollution; however, most of the research was conducted before the advent of modern high-throughput technologies, thus several gaps in the knowledge still need to be filled. Nowadays, omics approaches offer the possibility to address unsolved questions regarding the specific responses induced in plants by air pollution and may help in the selection of plants for the mitigation of air pollution. The establishment of next-generation sequencing (NGS) platforms has provided unprecedented tools for the analysis of DNA and RNA molecules, paving the way for the in-depth characterization of genomes, transcriptomes, epigenomes, and metagenomes [40]. Indeed, different from microarray technologies that require a reference genome and transcriptome for the experimental setup, NGS allows the de novo assembly of DNA and RNA sequences, significantly improves the accuracy of sequences, and enables the identification of small modifications in sequences, such as those induced during stress responses [41,42]. At the same time, the coupling of mass spectrometry (MS) with gas chromatography (GC-MS), ultra- and high-performance liquid chromatography mass spectrometry (UPLC-MS and HPLC-MS), matrix-assisted laser desorption/ionization-time-of-flight mass spectrometry (MALDI-TOF MS), and Fourier transform ion cyclotron resonance mass spectrometry (FT-ICR-MS), has provided powerful tools for the analysis of proteomes and metabolomes in different plant species, allowing the identification of variations in quantitative and qualitative traits in response to stress perception [43,44,45]. The use of an integrative approach based on genomics, proteomics, and metabolomics data has been recently proposed to identify and improve traits conferring increased abiotic stress tolerance in tomato (*Solanum lycopersicum* L.) plants [46].

So far, omics studies regarding the consequences of air pollution on plant growth have focused mainly on the effect of O_3_, NO_2_, and PM, which activate general stress responses common to abiotic and biotic stress in plants, such as the induction of ROS-scavenging enzymes [47,48,49]. Recent transcriptomics, proteomics, and metabolomics analysis has shed light on the responses induced in plants by air pollution, providing insightful cues for the identification of useful tolerance traits toward air pollution (Table 1). Indeed, different omics platforms provide insights on air pollution damage at multiple omics levels. While metabolomics and proteomics show differential abundance of specific molecules, determining a biochemical endpoint, transcriptomics revealed an impaired upstream regulatory mechanism.

Most of air pollutants enhance the formation of ROSs, such as hydrogen peroxide (H_2_O_2_), superoxide ion (O_2_^· −^), and hydroxyl radicals (OH^.^), and strongly activate the oxidative stress-responsive pathways. Among them, the expression and activity of ROS-scavenging enzymes often resulted in impairment, as well as the biosynthetic pathway of phenylpropanoids that is crucial for the synthesis of antioxidant phenolic compounds, such as flavonoids and phenolic acids. In the next sections, data from transcriptomic, proteomic, and metabolomic analysis carried out on plants exposed to O_3_, NO_2_, and PM are reviewed, unveiling key processes participating in the tolerance mechanisms implemented by plants to counteract air pollution.

### 2.1. Ozone

**Figure 2:**
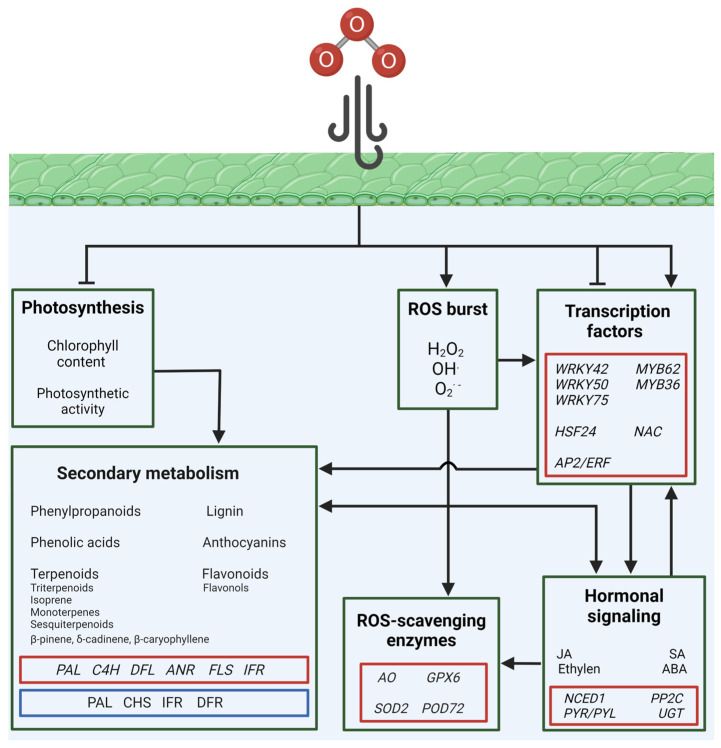
Schematic representation of the pathways affected by O_3_ (ozone) in plants. Sharp arrows and blunt arrows indicate induction and repression in pathways, respectively. Red squares enclose genes, while blue squares enclose proteins, affected by O_3_.

The effects and responses induced by O_3_ in plants have been extensively investigated in both model and non-model plant species, through fumigation experiments. Studies conducted on different accessions from *Arabidopsis thaliana* L. have provided significant information on the responses induced upon short-term exposure (2–6 h). Compared to control plants treated with 10–20 nL L^−1^ O_3_, treatments using 350–423 nL L^−1^ O_3_ revealed that the ecotype Columbia (Col) was tolerant to O_3_, while the ecotypes Shahdara (Sha) and Cape Verde Islands (Cvi) displayed significant signs of leaf damage, such as reduced photosynthetic performance and cell death [35]. Detrimental effects were observed in 50-day-old *Medicago truncatula* Gaertn. plants treated with 70 nmol mol^−1^ O_3_ for 6 h per day for 6 days, compared to controls grown using an environmental O_3_ concentration (~40 nmol mol^−1^). After fumigation with O_3_, *M. truncatula* showed a significant decrease in photosynthetic performance and an increase in ROS production [50]. Comparable results were obtained after exposing apple plants (*Malus* L. crabapple cv. Hongjiu) to 300 nL L^−1^ O_3_ for 3 h in an open-top chamber, which best simulates environmental conditions. The leaves from O_3_-treated plants displayed significant foliar damage and reduced chlorophyll content [51] (Figure 2). 

The transcriptomics data showed that Col, Sha, and Cvi shared the upregulation of several genes related to hormone signaling, including SA, JA, ethylene, and ABA, which are typically involved in responses induced by oxidative stress. In addition, O_3_ enhanced the expression of several members of the TF families, WRKY, AP2/ERF, and MYB, which are known to bind to the promoters of O_3_-responsive genes [35]. WRKY family TFs are involved in the stress-induced signaling cascade of JA and SA, AP2/ERFs participate in responsive mechanisms to various stresses, hormone signal transduction, and metabolite regulation, while MYBs have been shown to be key factors in the biosynthesis of secondary metabolites in plants, including anthocyanins, flavonols, and lignin, in response to multiple abiotic stresses [63,64,65]. Interestingly, *M. truncatula* plants exposed to O_3_ showed the upregulation of genes encoding for the transcription factors *WRKY42*, *WRKY50*, and *MYB62*, and genes related to JA signaling, while O_3_ induced the expression of *ERF* genes and *WRKY75* in apple plants [50,51]. Accordingly, metabolomic analysis also revealed that metabolites involved in the biosynthetic pathways of hormones were enriched in apple plants exposed to O_3_ [51]. These data confirm that, similarly to other stresses, *WRKY* expression and JA signaling cascades are closely connected during responses to air pollution stress (Figure 2). 

The tolerance traits identified in *A. thaliana* in response to short-term exposure to O_3_ included the regulation of the expression of genes encoding for H_2_O_2_ catabolism, such as *CAT* and *SOD*, that were downregulated in O_3_-sensitive Sha and Cvi [35]. On the other hand, increased SOD and POD activity was observed in O_3_-sensitive apple plants [51], suggesting the presence of other pathways participating in the tolerance mechanisms toward air pollution. Among the candidate pathways, that of flavonoids could play a pivotal role in tolerance to air pollution, as the flavonoid biosynthetic genes *phenylalanine ammonia-lyase* (*PAL*) and *cinnamate-4-hydroxylase* (*C4H*) were upregulated in the O_3_-tolerant Col [35]. However, also in O_3_-sensitive apple plants, the expression of *C4H*, *dihydroflavonol-4-reductase* (*DFR*), and *anthocyanidin reductase* (*ANR*), which code for pivotal enzymes in the flavonoid pathway, were upregulated, as well as the amount of anthocyanin and the metabolites involved in phenylpropanoid biosynthesis [51]. Altogether, these findings indicate that early responses to air pollution mainly involve the JA signaling cascade and the WRKY TF family. Additionally, fine tuning of the antioxidant machinery may confer tolerance traits to plants, depending on the regulation of the expression and activity of ROS-scavenging enzymes and the induction of the phenylpropanoid pathway. The timing and duration of these responses could also impact on air pollution tolerance (Figure 2). 

The effects of long-term exposure to high levels of O_3_ have been investigated both in controlled environments and in the open field. 

A recent study evaluated the tolerance to O_3_ using four rose cultivars (*Rosa hybrida* L. cv. “Schloss Mannheim”, “Iceberg”, “Lüye”, and “Spectra”) grown in open-top chambers and treated with unfiltered air, supplemented with 40 and 80 ppb O_3_, for up to 120 days. Out of the four cultivars, only Schloss Mannheim was sensitive to O_3_, showing detrimental effects, including foliar injury, reduced chlorophyll content, and a reduced net photosynthetic rate [52]. Similar results were observed in different legume crops, after 45 days of treatment with O_3_ concentration at about 151.2 nL L^−1^, in controlled conditions. Soybean (*Glycine max* L. Merr.) and common bean (*Phaseolus vulgaris* L.) plants exhibited negative physiological responses, such as reduced photosynthetic activity and damage on the leaves, while adverse effects were absent in garden pea (*Pisum sativum* L.) plants, indicating that these species have mechanisms that promote tolerance to O_3_ [53] (Figure 2). 

In the rose cultivar Schloss Mannheim compared to the others, the expression of several *heat stress transcription factors* (*HSFs*), which mediate responses to several abiotic stresses [66], such as *HSF24*, and the TFs *WRKY42*, *WRKY75*, *MYB36*, and *MYB62*, were upregulated by the exposure to O_3_ [52]. In addition, the expression of the ABA-related genes *NCED1*, *PP2Cs*, *PYR/PYL*, and *UGTs* were increased in Schloss Mannheim, sustaining the hypothesis that during O_3_ stress, ABA acts as a developmental signal integrating responses from different pathways [52]. Interestingly, upon O_3_ treatment, hormone metabolism was affected similarly in all three legume species; conversely, the transcription of several WRKY and MYB TFs was upregulated in the O_3_-tolerant garden pea, while their expression was negligible in the O_3_-sensitive soybean and common bean plants [53]. Long-term exposure to high levels of O_3_ induces the upregulation of the phenylpropanoid and flavonoid biosynthetic pathways in the rose cultivar Schloss Mannheim, in garden pea, soybean, and common bean plants [52,53]. These pathways are involved in the synthesis of different antioxidant molecules, such as phenolic acids, which contribute to the defense mechanisms against oxidative stress. Key enzymes for phenylpropanoid metabolism, including PAL, chalcone synthase (CHS), isoflavone reductase (IFR) and DFR, showed increased transcript abundance in all three legume species compared to control plants (O_3_ concentration ~12.5 nL L^−1^) and, accordingly, the phenolic content in leaves increased upon O_3_ treatment [53]. Interestingly, soybean and common bean plants exposed to O_3_ showed increased expression of *ascorbate oxidase* (*AO*), which negatively affected the amount of ascorbate, thus reducing the amount of active antioxidant molecules. Accordingly, the expression of genes encoding for ROS-scavenging enzymes was mainly unaffected in garden pea plants, while *glutathione peroxidase 6* (*GPX6*) and *SOD2* transcript levels increased in the soybean and common bean plants, indicating an active oxidative stress response in the latter plant species [53]. Altogether, these data suggests that precise and integrated molecular responses are activated to achieve tolerance to O_3_ and that even a few alterations to these mechanisms may trigger sensitivity to air pollution. On the other hand, Schloss Mannheim roses showed the induction of sesquiterpenoid and triterpenoid biosynthesis upon O_3_ treatment [52], suggesting that stressed plants may affect the level of air pollution. Indeed, although isoprene, monoterpenes, and higher terpenoids rapidly react with O_3_ in the atmosphere, likely reducing the O_3_ concentration, they may trigger the production of secondary air pollutants [67] (Figure 2).

A field study on coniferous *Abies religiosa* ([Kunth] Schlechtendahl et Chamisso) trees grown in an area sensitive to O_3_ contamination in Mexico City (Mexico), evaluated the effect of O_3_ on leaves during different periods of the year. Each timepoint was characterized by different O_3_ concentrations, corresponding to moderate (87 ppb), intermediate (120–94 ppb), and high (170 ppb) concentrations. Histologic, metabolomic, and transcriptomic analysis revealed that within individuals from the same plant species, different responses were visible [54]. Symptomatic leaves showed a thicker epidermis and collapsed cells in the palisade parenchyma compared to asymptomatic leaves. The expression of several TFs of the *NAC* family, commonly involved in multiple stresses such as drought, high salinity, and in ABA and JA signaling [68], was upregulated in symptomatic trees, as well as the expression of *peroxidase 72* (*POD72*). On the contrary, genes encoding for flavonoids, such as *flavonol synthase* (*FLS*) and *IFR*, were downregulated in symptomatic trees. Interestingly, the expression of genes related to terpene biosynthesis and BVOC emissions was induced in asymptomatic trees. Metabolomic analysis confirmed deregulation in terms of the genes involved in terpene metabolism, showing significant differences in terpene composition among individuals, particularly in sesquiterpenes, such as β-pinene, δ-cadinene, and β-caryophyllene, that were induced in asymptomatic trees. It is probable that sesquiterpenes contributed to the degradation of ROSs and were higher in asymptomatic trees [54]. 

Overall, the fast induction of ROS detoxifying machinery highlighted its key role as a first-line defensive response to air pollutants in plants. Additionally, the general upregulation in antioxidant molecules, such as phenylpropanoids, was observed in response to O_3_. However, plants sensitive to air pollution showed significant impairment in these mechanisms, eventually leading to adverse symptoms (Figure 2). 

### 2.2. Nitrogen Dioxide

Increasing concerns related to air quality in urban areas are being raised due to the high amount of nitrogen dioxide (NO_2_) generated by anthropic activities. However, few studies have investigated the impact of this molecule on plant development, with most of studies focusing on injury symptoms, physiological effects, and photosynthetic performance [55]. 

**Figure 3:**
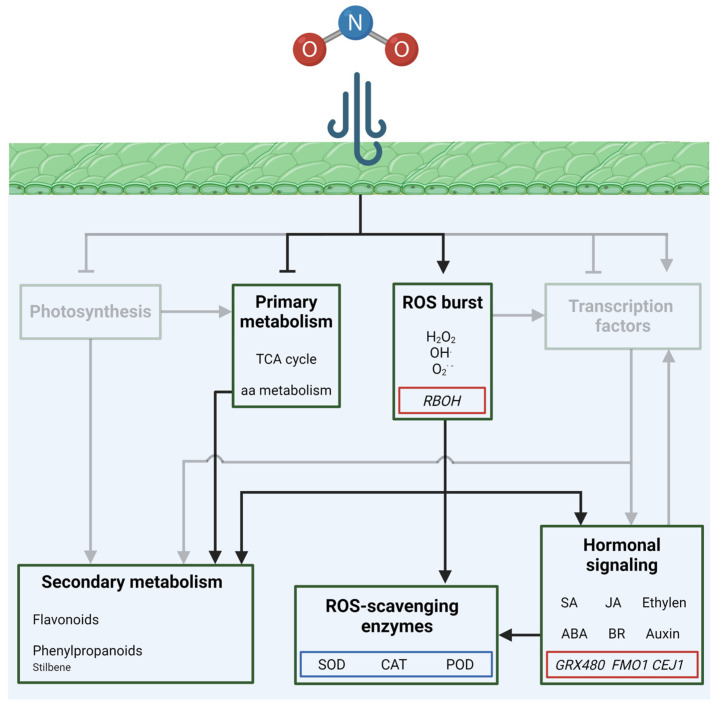
Schematic representation of the pathways affected by NO_2_ (nitrogen dioxide) in plants. Sharp arrows and blunt arrows indicate induction and repression in pathways, respectively. Red squares enclose genes, while blue squares enclose proteins, affected by NO_2_. Pathways that are not primarily involved are shaded.

Details regarding the effects of air pollution on gene expression in plants were provided by an in-depth study on approximatively 372 different accessions from *A. thaliana* plants treated with up to 30 ppm of NO_2_ for 1 h, or up to 400 ppm of O_3_ for 2–6 h [56]. Depending on the accession, the plants displayed different degrees of tolerance to O_3_ and NO_2_, which were investigated by a genome-wide association study (GWAS). A comparison of transcriptomic and microarray data revealed that O_3_ and NO_2_ induced similar responses, which included the expression of genes involved in hormone signaling [56]. Transcripts for marker genes related to JA and ethylene signaling, such as those *cooperatively regulated by ethylene and jasmonate 1* (*CEJ1*), and those related to SA signaling, like *glutaredoxin 480* (*GRX480*) and *flavin-dependent monooxygenase 1* (*FMO1*), were significantly more abundant in plants treated with NO_2_ and O_3_ compared to the controls [56]. Transcriptomic analysis performed on pollen from common ragweed (*Ambrosia artemisiifolia* L.) exposed to long-term fumigation (61 days) with 40 ppb (control) and 80 ppb NO_2_ (treatment), as well as 40 ppb (control), 80 ppb, and 120 ppb O_3_ (treatments), showed significant enrichment in gene ontology (GO) terms related to the response to abiotic and biotic stress, JA biosynthetic processes, and phosphate cell homeostasis [57]. Furthermore, in treated pollen GO terms, including the response to ethylene stimulus, ABA and auxin signaling pathways were enriched, mainly in upregulated transcripts. These findings highlight the prominent role of phytohormones in response to air pollution, in particular that of JA signaling, which has a key role in response to NO_2_ and O_3_ [57] (Figure 3).

Both NO_2_ and O_3_ induced genes involved in ROS production and metabolism, although differences were observed among the two gases. The expression of *respiratory burst oxidase homolog F* (*RBOH*), which encodes for an NADPH oxidase involved in ROS synthesis, was upregulated upon O_3_ treatment, while it decreased after NO_2_ treatment [56]. However, the induction of oxidative stress by NO_2_ is well established. Recent research on *Bougainvillea spectabilis* Willd. seedlings exposed to short-term high-concentration fumigation, with up to 8 μL⋅L^−1^ NO_2_ for 8 h, showed the induction of yellow–brown spotting on the leaves, which was likely related to oxidative stress [55]. Indeed, POD, SOD, and CAT activity was significantly increased in seedlings treated with NO_2_ compared to the controls, suggesting the activation of ROS-induced stress responses [55]. In addition, metabolomic analysis highlighted significant differences in the metabolites related to flavonoid and stilbene biosynthesis, amino acid metabolism, and the tricarboxylic acid (TCA) cycle, among treated and control plants, supporting the hypothesis that increased oxidative stress occurs upon NO_2_ exposure in *B. spectabilis* [55] (Figure 3).

Interestingly, among the effects induced in plants by NO_2_ treatment, there is a significant increase in pollen allergen transcript amounts, which could pose risks for human health. On the other hand, a significant decrease in allergen transcripts was found in pollen treated with high levels of O_3_, supporting the notion that NO_2_ and O_3_ regulate the expression of the same genes in an opposite way [57].

### 2.3. Particulate Matter and Heavy Metals

Among the air pollutants found in highly urbanized areas, PM potentially poses a high-risk to health, thus deciphering how plants cope with this stress could provide insightful information to identify tolerant plants, which may help in mitigating air pollution.

**Figure 4:**
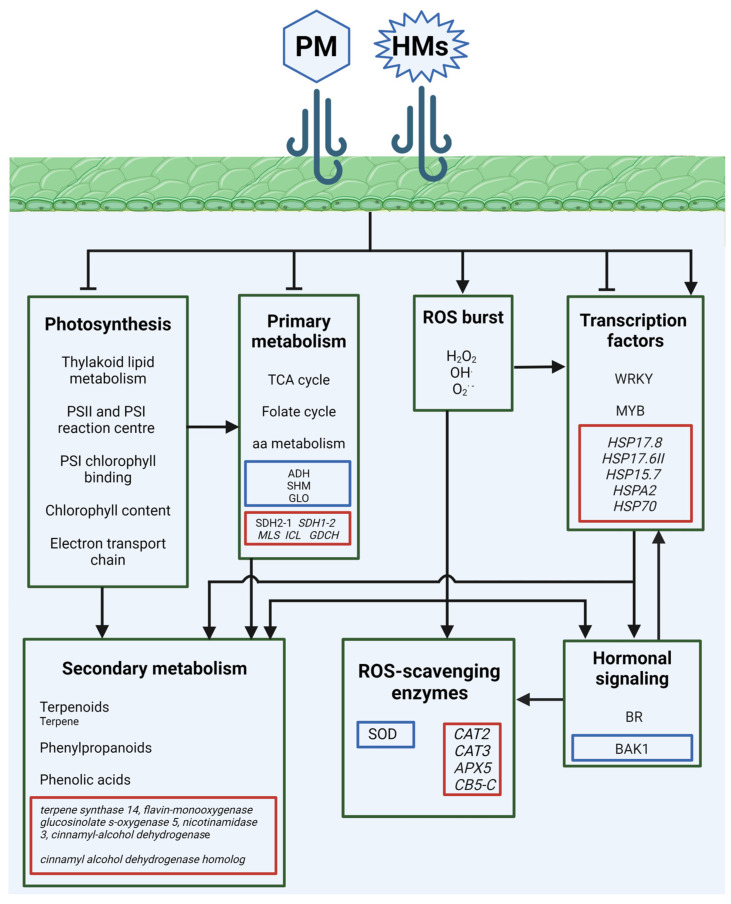
Schematic representation of the pathways affected by PM and PM-containing HMs (particulate matter, heavy metals) in plants. Sharp arrows and blunt arrows indicate induction and repression in pathways, respectively. Red squares enclose genes, while blue squares enclose proteins, affected by PM and HMs.

New data on plant responses to PM were obtained through the exposure of the ornamental plant species *Wrightia religiosa* (Teijsm. & BINN.) Hook. F. and *Sansevieria trifasciata (Dracaena trifasciata* Prain.) to burning cigarettes as a source of pollution [58,59,60]. The plants were placed in an enclosed chamber and exposed to a concentration of PM_1_, PM_2.5_, and PM_10_ of up to 900–945, 900–945, and 950–980 μg m^−3^, respectively, for about 7 days. Interestingly, priming the plant, i.e., the attitude of a plant exposed to a certain stress, to better tolerate subsequent stress treatments [69], to PM stress was also investigated [58,59,60]. Proteomic analysis revealed that in *W. religiosa* most of the proteins related to photosystem II (PSII), the photosystem I (PSI) reaction center, and PSI chlorophyll binding, were downregulated. Consistently, leaf chlorophyl content decreased after the treatment [58]. On the other hand, in *S. trifasciata* plants exposed to cigarette smoke, photosynthesis performance was unaffected, and an upregulation of the proteins involved in PSI and PSII assembly was observed [59]. In detail, the leaves of treated *S. trifasciata* plants showed specifically expressed unique proteins involved in biological processes related to photosynthesis, chlorophyll binding, and electron transport chains, which were not identified in control leaves, suggesting specific activation of the photosynthetic process in *S. trifasciata* exposed to air pollution [59]. Accordingly, a recent transcriptomic analysis performed on leaves from the ornamental shrub *Photinia × fraseri* Dress. and *Laurus nobilis* L. grown for three months in a rural area and near to a busy road in the city of Altopascio (Lucca, Italy), showed a significant deregulation in genes encoding for PSI and PSII assembly machinery in response to air pollution [61,62]. *P. fraseri* and *L. nobilis* plants grown along the road, characterized by an average concentration of PM_10_ and PM_2.5_ in the air of 10.63 and 6.05 μg m^−3^, respectively, showed significant impairment in key genes involved photosynthesis, as well as in those involved in the TCA cycle. The expression of succinate dehydrogenase [ubiquinone] iron–sulfur subunit 1 (*SDH2–1*), succinate dehydrogenase [ubiquinone] flavoprotein subunit 2 (*SDH1–2*), malate synthase (*MLS*), isocitrate lyase (*ICL*), and glycine decarboxylase complex (*GDCH*), was downregulated in plants exposed to a high PM concentration. A clear downregulation was observed for genes related to desaturase enzymes, including the fatty acid desaturase family protein, 16:0delta9 desaturase 2, delta 9 desaturase 1, and Acyl-coenzyme a desaturase-like2, which play pivotal roles in thylakoid lipid metabolism and could affect the photosynthetic machinery of both *P. fraseri* and *L. nobilis* [61,62] (Figure 4).

Particulate matter also affected BR pathways in *P. fraseri*, *L. nobilis,* and *S. trifasciata*, as demonstrated by the upregulated expression of genes and the increased abundance of proteins related to BR signaling, such as brassinosteroid-insensitive 1-associated receptor kinase 1 (BAK1), which is involved in the repression of the production of ROSs through the stimulation of antioxidant activity [59,61,62]. On the other hand, there was no clear differential modulation of any class of hormone-related genes; however, several TFs involved in plant development, stress responses, and JA signaling, including MYBs and WRKYs, were deregulated in *P. fraseri* and *L. nobilis* in response to PM stress [61,62] (Figure 4).

Proteomic analysis of *S. trifasciata* plants fumigated with PM showed that oxidative stress was negligible in this plant species, as the amount of the ROS-scavenging enzyme was mainly unaffected, except for that of SOD, which decreased after PM treatment [59]. On the other hand, genes encoding for several enzymes involved in the biosynthesis of phenylpropanoids and phenols, such as the *cinnamyl alcohol dehydrogenase homolog*, were downregulated in both *P. fraseri* and *L. nobilis* grown in the urban area. Conversely, plants from the rural area, characterized by an average concentration of PM_10_ and PM_2.5_ in the air of up to 12.11 and 8.43 μg m^−3^, respectively, showed an upregulation of genes involved in terpene and phenylpropanoid-related pathways, such as *terpene synthase 14*, *flavin-monooxygenase glucosinolate s-oxygenase 5*, *nicotinamidase 3* and *cinnamyl alcohol dehydrogenase*, as well as an upregulation of genes encoding for *cytochrome B5 isoform C* (*CB5-C*), *CAT2*, *CAT3*, *peroxiredoxin type 2*, and *APX5*. These data suggest that ROS-scavenging enzymes are more effective in rural areas rather than urban centers. However, ROS scavenging could be an early stress response to a high PM concentration, thus not being detected in plants exposed to PM in the long term. The expression of *heat shock proteins* (*HSPs*), which act downstream of HSFs and regulate the response to stress [70], was mainly induced in plants grown in the urban area, and genes encoding for *HSP17.8*, *HSP17.6II*, *HSP15.7*, *HSPA2*, and *HSP70* were all upregulated, compared to plants grown in the rural area. Interestingly, pathogenesis-related genes, such as the *putative pathogenesis-related thaumatin superfamily protein* (*ATLP-1*) and *putative basic pathogenesis-related protein 1* (*ATPRB1*) and the *disease resistance* protein (*Q19e69*) were induced in *P. fraseri* plants grown in high PM conditions [61]. Similar results were obtained for *A. thaliana* plants exposed to 30 mg m^−3^ of SO_2_, which showed upregulation of generic HSPs and pathogen-related proteins [48,49] (Figure 4).

A recent innovative protocol allowed the low-cost extraction of both the soluble and insoluble fractions of PM_2.5_, which were tested in *A. thaliana* seedlings grown for 14 days on a medium with up to 5 g L^−1^ of PM added. Treatment with both soluble and insoluble PM_2.5_ induced a significant decrease in chlorophyl content and enhanced oxidative stress due to the superoxide anion (radical O_2_^· −^) increase in plants. Analysis of PM revealed the presence of chemical elements such as As, Cd, and Cobalt (Co), which were accumulated in treated seedlings [71]. Accordingly, GO classification identified differentially abundant proteins (DAPs) involved in the responses to metal ion and cadmium ion stimuli in *S. trifasciata* plants fumigated with PM [59]. This result is consistent with previous reports on the presence of HMs in cigarette smoke, which is especially rich in Cd (1–2 µg g^−1^) [72]. In *S. trifasciata* leaves from treated plants, the activation of an alternative carbon metabolism was found. The modulation of alcohol dehydrogenase (ADH), serine hydroxymethyltransferase (SHM), and glycolate oxidase (GLO) enzymes, which allow organic compound assimilation through the folate cycle to produce serine, probably enabled *S. trifasciata* to use the absorbed PM as a carbon source [59] (Figure 4).

Upon treatment with cigarette smoke, the relative water content was found to be increased in *W. religiosa* leaves, in agreement with the observed upregulation of the putative homolog of the aquaporin-related gene TIP2-2 in *S. trifasciata*, suggesting a probable increase in the water channel amount in response to air pollution [58,59,60]. Altogether, these data support the notion that a general response to abiotic stress is activated upon exposure to PM, O_3_, and NO_2_. The identification of the traits for air pollution tolerance in plants is at the beginning and further research is needed to disentangle the network involving JA signaling, WRKY and MYB TFs, ROS-scavenging enzymes, and antioxidant biosynthesis, especially that of phenylpropanoids and flavonoids, in response to air pollution.

## 3. Conclusions

**Figure 5:**
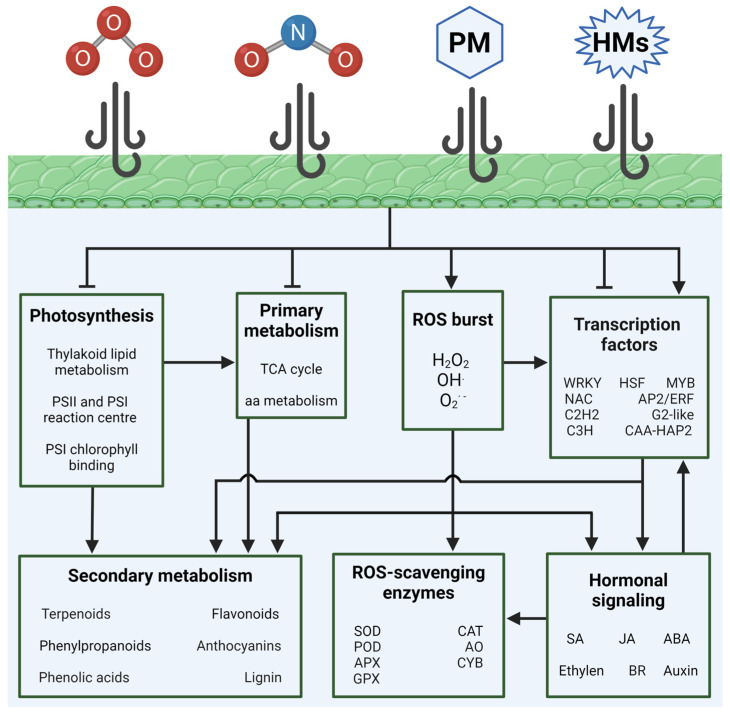
Schematic diagram of the pathways affected by the most common urban air pollutants in plants. Sharp arrows and blunt arrows indicate induction and repression in pathways, respectively.

The increase in air pollution is increasing the risks associated with human and environmental health. The development of NBSs centered on the greening of urbanized regions could be a sustainable strategy to mitigate air pollution. Unveiling the molecular mechanisms that promote plant tolerance to air pollution could provide useful insights in order to select appropriate plant species for strategies aimed at the improvement of air quality. Common pathways were induced in response to treatments using different air pollutants, such as the upregulation of ROS-scavenging enzyme activities and gene expression in sensitive plants. The upregulation of antioxidant molecules, like flavonoids, was confirmed by transcriptomic, proteomic, and metabolomic analysis in different plant species, both sensitive and tolerant to air pollution stress. These data suggest a prominent role for phenylpropanoid metabolism in plant tolerance to air pollution. The recurring involvement of AP2/ERF, WRKY, and MYB TFs families was observed, along with different plant species, as well as the impairment of phytohormone signaling, including that of ABA, JA, and ethylene (Figure 5).

Altogether, these data provide crucial tools for the evaluation of tolerance traits in plants toward several molecules that affect air quality, such as O_3_, NO_2_, and PM, providing the basis for a detailed understanding of responses induced by air pollution stress.

## Figures and Tables

**Table 1:** Summary of omics techniques and affected pathways induced by air pollution in plant species.

Species	Experimental Setup	Pollutant	Exposure	Omics Platform	Enriched Pathways	Reference
*Arabidopsis* *thaliana* L.	Controlled environment growth chambers	O_3_ 350–423 nL L^–1^	2–6 h	Transcriptomics (RNA-seq)	Photosynthesis Response to SA Response to ROS Response to JA Response to ethylene ABA signaling pathway	[35]
*Medicago* *truncatula* L.	Controlled environment growth chambers	O_3_ 70 nmol mol^−1^	6 h d^−1^ for 6 d	Transcriptomics (microarray)	Phenylalanine biosynthesis Sugar metabolism Photosynthetic electron transport Responses to inorganic substances	[50]
*Malus* L.	Open-top growth chamber	O_3_ 300 nL L^−1^	3 h	Transcriptomics (RNA-seq) Metabolomics (UPLC MS/MS)	Chloroplast thylakoid membrane Chloroplast photosystem I H_2_O_2_ dehydratase activity Chalcone synthase activity Flavonoid metabolism Hormone pathways	[51]
*Rosa* *hybrida* L.	Controlled environment growth chambers	O_3_ 80 ppb	10 h	Transcriptomics (RNA-seq)	Phenylpropanoid biosynthesis Starch and sucrose metabolism Sesquiterpenoid biosynthesis Triterpenoid biosynthesis	[52]
*Pisum sativum* L. *Glycine max* L. *Phaseolus vulgaris* L.	Controlled environment growth chambers	O_3_ ~151.2 nL L^−1^	8 h d^−1^ for 45 d	Transcriptomics (RNA-seq)	Phenylpropanoid metabolism Ascorbate–glutathione cycling Glycolysis TCA cycle	[53]
*Abies religiosa* Schltdl. & Cham.	Urban environment	O_3_ 87–170 ppb	3 years	Transcriptomics (RNA-seq)	Carbohydrate metabolism Gene regulation Transcription factors Defense regulation Terpenes	[54]
*Bougainvillea* *Spectabilis* Willd.	Controlled environment growth chambers	NO_2_ 8 μL L^−1^	8 h	Metabolomics (UPLC-MS)	Biosynthesis of amino acids Phenylalanine metabolism Phenylpropanoid biosynthesis Starch and sucrose metabolism Glutathione metabolism TCA cycle	[55]
*Arabidopsis* *thaliana* L.	Controlled environment growth chambers	O_3_ 350 ppb NO_2_ 10 ppm	2 h O_3_ 1 h NO_2_	Transcriptomics (RNA-seq and microarray)	Pathogen resistance Cell death Ethylene signaling	[56]
*Ambrosia* *artemisiifolia* L.	Controlled environment growth chambers	O_3_ NO_2_ 40–80 ppb	61 d	Transcriptomics (RNA-seq)	Jasmonic acid pathway Response to ethylene stimulus Response to auxin stimulus Abscisic acid signaling pathway	[57]
*Wrightia religiosa* (Teijsm. & BINN.) Hook. F.	Controlled environment growth chambers	PM_2.5_ 470–500 μg m^−3^ cigarette smoke-derived	24 h	Proteomics (LC-MS/MS)	Photosynthetic proteins	[58]
*Sansevieria trifasciata (Dracaena trifasciata* Prain.)	Controlled environment growth chambers	PM_1_ up to 945 μg m^−3^, PM_2.5_ up to 945 μg m^−3^, PM_10_ up to 980 μg m^−3^ cigarette smoke-derived	24 h	Proteomics (LC-MS/MS) Metabolomics (LC-MS/MS)	Precursor metabolites Photosynthesis Alternative carbon metabolism Brassinosteroid signaling Stress-related proteins Metal and cadmium ion stimuli	[59,60]
*Photinia* *× fraseri* L.	Urban environment	PM_2.5_, PM_10_ traffic-derived 12.11 and 10.63 μg m^−3^	3 months	Transcriptomics (RNA-seq)	Leaf primary metabolism Biotic stress response Abiotic stress response Cell cycle and cell division Transcription factors	[61]
*Laurus* *nobilis* L.	Urban environment	PM_2.5_, PM_10_ traffic-derived 12.11 and 10.63 μg m^−3^	3 months	Transcriptomics (RNA-seq)	Primary metabolism Secondary metabolism Hormone-related pathways Environmental stress response Transcription factors	[62]
